# Sorting nexin 10 acting as a novel regulator of macrophage polarization mediates inflammatory response in experimental mouse colitis

**DOI:** 10.1038/srep20630

**Published:** 2016-02-09

**Authors:** Yan You, Chun Zhou, Dong Li, Zhong-Lian Cao, Weixing Shen, Wan-Zhen Li, Sulin Zhang, Bin Hu, Xiaoyan Shen

**Affiliations:** 1 Department of Pharmacology, School of Pharmacy, Fudan University, Shanghai, China; 2 Laboratory of Pharmacology and Toxicology, School of Pharmaceutical Sciences, Sun Yat-sen University, Guangzhou, China; 3 The Translational Medicine Research Center, Nanjing University of Traditional Chinese Medicine, Nanjing, China

## Abstract

In response to changes in microenvironment, macrophages polarize into functionally distinct phenotypes, playing a crucial role in the pathogenesis of inflammatory bowel disease (IBD). Here, we investigated the effects of sorting nexin 10 (SNX10), a protein involved in endosomal trafficking and osteoclast maturation, on regulation of macrophage polarization and progression of mouse colitis. Our results revealed that SNX10 deficiency increased the population of M2-type monocytes/macrophages, and protected against colonic inflammation and pathological damage induced by dextran sulfate sodium (DSS). By *in vitro* study, we showed that deficiency of SNX10 polarized macrophages derived from mouse bone marrow or human peripheral blood mononuclear cells (PBMCs) towards an anti-inflammatory M2 phenotype, which partially reversed by SNX10 plasmid transfection. Adoptive transfer of SNX10^−/−^ macrophages ameliorated colitis in WT mice. However, transfer of WT macrophages exacerbated colitis in SNX10^−/−^ mice. Our data disclose a crucial role and novel function for SNX10 in macrophage polarization. Loss of SNX10 function may be a potential promising therapeutic strategy for IBD.

Macrophages are the most abundant mononuclear phagocytes in the healthy intestinal lamina propria. They are situated at the interface of intestinal lumen to exert appropriate responses to microbes and ingested stimuli, and play an essential role in the maintenance of tissue homeostasis^1^. Dysfunction of macrophage results in an excessive response to intestinal microbial stimuli, inefficient clearance of microbes from host tissues, and impaired proper transition from pro- to anti-inflammatory responses, contributing to the pathogenesis of inflammatory bowel disease (IBD)^1^,^2^. In response to changes in microenvironment, macrophages polarize into functionally distinct phenotypes: classically activated (M1) macrophages and alternatively activated (M2) macrophages. M1 polarization is induced by IFN-γ, lipopolysaccharide (LPS), and TNF-α, and results in increased production of pro-inflammatory cytokines (such as IL-23, TNF-α, and IL-6) and reactive oxygen species. These mediators promote the differentiation and activation of Th1 and Th17 cells, and further aggravate the inflammation and tissue damage^3^. M2 polarization is stimulated by IL-4, IL-13, and M-CSF, and leads to low levels of pro-inflammatory cytokines and higher expressions of IL-10, inflammatory zone 1 (FIZZ1), mannose receptor (CD206), arginase I (Arg1), and CD163 antigen^4^,^5^. Unlike the pro-inflammatory M1 macrophage, the M2 macrophage is anti-inflammatory and participates in tissue restitution after injury^5^. Therefore, macrophage polarization plays a crucial role in the onset, progression, and prognosis of inflammation. An imbalance of M1 and M2 macrophages has been implicated in the development of experimental IBD^6^,^7^,^8^.

Sorting nexins (SNXs) are phosphoinositide- binding proteins containing a phox homology-domain, through which SNXs are targeted to endosomal membrane and function in a variety of endosomal transport pathway^9^,^10^. As one of the simplest structural members of the SNX family, SNX10 has been reported to possess a regulatory function in endosomal/lysosomal pathway^11^, which is crucial for the functions of macrophages, including phagocytosis and digestion of pathogens, antigen processing and presentation, inflammatory response, as well as the maintenance of tissue homeostasis^12^,^13^. Recently, we found that SNX10 is required for osteoclast maturation and function, deficiency of SNX10 is associated with a diminished production of systemic pro-inflammatory cytokines^14^. As patients with IBD are at a greater risk for excessive bone loss of which the precise mechanisms are not yet completely understood, it inspired us to explore the potential role of SNX10 in the macrophage function and pathogenesis of IBD by directly connecting inflammation with osteoclast activation. In the present study, we report that SNX10 knockout effectively protected against inflammation and pathological damage in dextran sulfate sodium (DSS)-induced mouse colitis through promoting M2 polarization of macrophages. Our results disclose a novel function of SNX10 in regulating macrophage polarization and function, and indicate that SNX10 is a promising therapeutic target for IBD and its complication treatment.

## Materials and Methods

### Mice and colitis induction

SNX10^−/−^ homozygous and littermate mice background in FVB were transferred from the Institute of Development Biology and Molecular Medicine, Fudan University, Shanghai, China. Male SNX10^−/−^ homozygous mice and their wild type (WT) littermate mice aged 8 to 10-weeks received 3% (wt/vol) dextran sulfate sodium (DSS, 36–50 kDa, MP Biomedicals, Santa Ana, CA, USA) dissolved in drinking water for 7 days^15^. Control mice received regular drinking water throughout the experiment. Animal weight was recorded daily. Mice were euthanized at day 8, and samples were collected. All animal procedures were performed following the “Guide for the Care and Use of Laboratory Animals” published by the National Institutes of Health (NIH) and were approved by the ethics committee of Experimental Research, Shanghai Medical College, Fudan University.

### Bone-marrow-derived macrophages (BMDMs) preparation and treatment

BMDMs were derived from WT and SNX10^−/−^ mice as described by Manicone *et al.*^16^. For rescue experiments, SNX10^−/−^ macrophages were transfected with Ad-vector or Ad-SNX10 at day 6 of induction for 24 h. For M1-like polarization, macrophages at day 7 were stimulated with 1 μg/ml LPS (Sigma-Aldrich, St. Louis, MO, USA); for M2 polarization, cells were treated with 20 ng/ml IL-4 (PeproTech, Rocky Hill, NJ, USA). The cells were then harvested and were characterized by RT-real time PCR for Arg-1, FIZZ1, CD206 (M2-type genes), and also TNF-α, IL-6 and IL-10.

### Preparation and treatment of human macrophages

Peripheral blood mononuclear cells (PBMCs) were isolated from heparinized peripheral blood of healthy voluntary donors with Human Lymphocyte Separation tube (Dakewe Biotech Co., LTD, China), and then were differentiated into macrophages for 7d in complete RPMI medium containing 20 ng/ml recombinant human M-CSF (PeproTech, Rocky Hill, NJ, USA).

Small interference RNA (siRNA) targeting human SNX10 (5′-CAGGGCTTGGAAGATTTCCTCAGAA), and Silencer Negative control siRNA (NC), which does not match any sequence in the human genome, were from Invitrogen. Lipofectamine 3000 was used for siRNA transfection according to the manufacturer’s instructions (Invitrogen, Grand Island, NY, USA). Human macrophages treated with control and SNX10 siRNA were then stimulated with 20 ng/ml human IL-4 (PeproTech, Rocky Hill, NJ, USA) for 24 h or 1 ug/ml LPS for 6 h. The study was approved by the Fudan University Hospital Ethics Committee.

### *In vivo* depletion of macrophages and adoptive transfer

The suicidal liposome technique has been used to deplete macrophages^17^. Briefly, mice (6 in each group) received 100 μl clodronate or control PBS-loaded liposomes via caudal vein injection at day 1 and 2. On the day 3, SNX10^−/−^ mice and their WT littermates were transplanted with BMDMs (1.0 × 10^6^ BMDMs per mouse) derived from WT or SNX10^−/−^ mice^18^. Colitis was induced 24 hours after transfer of the macrophages. Macrophages were labeled *in vitro* with red fluorescent dye IR783B-NHS (a gift from Professor Cong Li in the School of Pharmacy, Fudan University, Shanghai, China).

### Isolation of colonic lamina propria macrophages

Lamina propria macrophages were isolated using a modified protocol as described^19^. Briefly, dissected large intestines were weighed and cut into 2-mm pieces. After washing with iced Hanks’ balanced salt solution (HBSS), the intestinal tissue samples were digested with 0.25 mg/ml of collagenase (Sigma-Aldrich, St. Louis, MO, USA), 1 mg/ml dispase (Invitrogen, Grand Island, NY, USA USA) in HBSS containing 25 U/ml DNase I (Roche Diagnostics, Indianapolis, IN, USA), 1 M CaCl_2_ and 10% fetal calf serum at 37 ^o^C for 30 min. The extracted cells were filtered and analyzed by flow cytometry.

### Flow cytometry

Cell phenotypic characteristics were determined using flow cytometry. Macrophages from colon were isolated and then stained with PE-conjugated F4/80, APC-conjugated CD206 (eBiosciences, San Diego, CA, USA). CD206^+^ F4/80^+^ macrophages were identified as M2-type Macrophages. Peripheral blood mononuclear cells were stained with FITC-labeled anti-Ly6C (Biolegend, San Diego, CA, USA), and PE-labeled anti-CX3CR1 (R&D Systems, Minneapolis, MN, USA) antibodies. Ly6C was used to gate on CX3CR1-low cells to identify the Ly6C^high^CX3CR1^low^ population (M1 type monocytes), or gate on CX3CR1-high cells to identify the Ly6C^low^CX3CR1^high^ population (M2 type monocytes). Nonspecific binding was blocked with Fc block (BD Biosciences, San Jose, CA, USA). At least 1×10^5^ events were collected per sample.

### Statistics

Statistical analyses were performed using SPSS 13 (Statistical Program for Social Sciences). The Independent-Samples T Test or nonparametric Mann-Whitney U test were used to compare the two groups, and a one-way ANOVA followed by Bonferroni post hoc test for multiple comparison was used. *P* < 0.05 was the significance level. All the numerical data presented are representative of at least 3 repeat experiments and are expressed as mean ± SEM.

## Results

### SNX10 deficiency ameliorates DSS-induced colitis in mice

**Figure 1:**
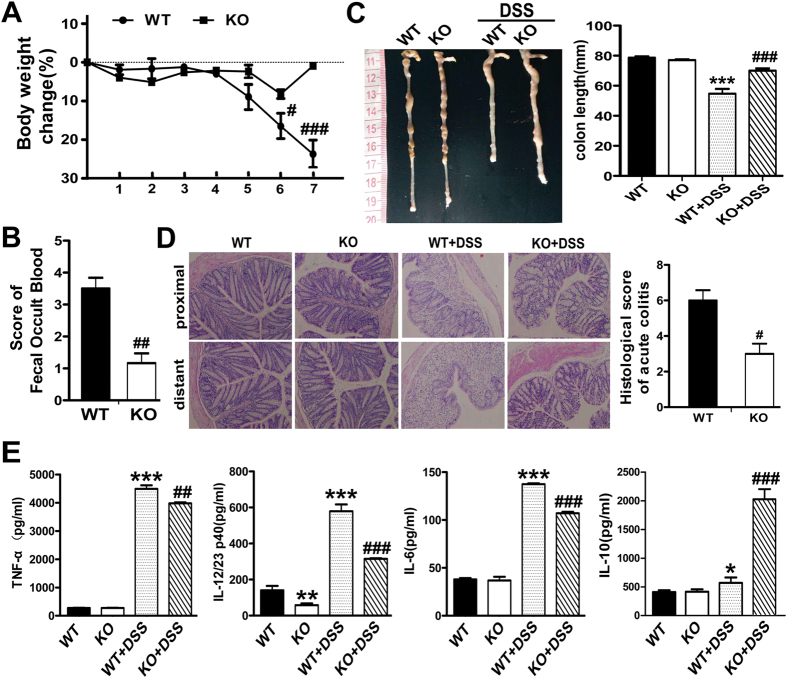
SNX10 deficiency ameliorates DSS-induced colitis in mice. SNX10-deficient mice (KO) and littermate wild type mice (WT) were fed with 3% DSS for 7 days. (**A**) Weight loss was measured daily and expressed as the average percentage of initial body weight ± SEM. (**B**) Blood contents in stools of WT and SNX10 KO were scored on the 7^th^ day. (**C**) The length of colons from WT or SNX10 KO mice was measured at day 7 after euthanasia. (**D**) Representative histological images (×20) and the scores of colonic inflammation of SNX10 KO and WT mice on the 7^th^ day were compared. (**E**) The concentration of TNF-α, IL-12/23p40, IL-6 and IL-10 in the serum of WT and SNX10 KO mice with or without DSS induction were measured by ELISA. **P < 0.01, ***P < 0.001 vs WT normal control group; ^#^P < 0.05, ^##^P < 0.01, ^###^P < 0.001 vs WT with DSS induction group; n = 10 in each group.

To explore the possible role of SNX10 in IBD, we compared the severity of DSS-induced colitis in SNX10^−/−^ and WT mice. In SNX10^−/−^ mice, the loss of body weight observed in WT mice was significantly alleviated from 6 to 8 days after DSS administration (Fig. 1A); consistently, the fecal occult blood score was reduced in SNX10 deficient mice as compared to WT mice (Fig. 1B). In WT mice, DSS treatment resulted in a marked reduction of colon length, which was improved significantly by SNX10 deficiency (Fig. 1C). Histological assessment showed inflammatory cell infiltration, severe epithelial destruction and crypt loss in the colon of WT mice at day 7 after DSS administration, whereas in SNX10^−/−^ mice, those characteristics were improved (Fig. 1D), and the histological score of acute colitis was significantly lower than that of WT mice (Fig. 1D). Accordingly, the concentrations of TNF-α, IL-6, and IL-12/23 in serum were increased in DSS-treated WT mice, but were much lower in DSS-treated SNX10^−/−^ mice in which IL-10 was significantly increased (Fig. 1E). Interestingly, even in the normal condition, SNX10 deficient mice have lower levels of IL-12/23 in serum (Fig. 1E). These findings provide evidence that SNX10 closely relates to inflammatory responses of colitis.

### SNX10 deficiency increases the population of M2-type monocytes and macrophages

**Figure 2:**
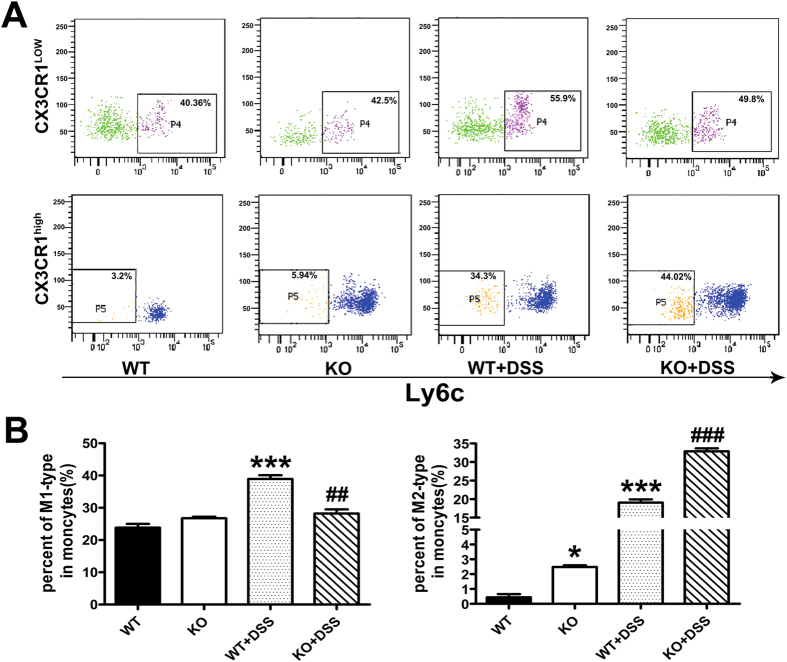
SNX10 deficiency increases the population of M2-type monocytes in peripheral blood. (**A**) Representative flow cytometry analysis of monocyte populations in peripheral blood isolated from WT and SNX10 KO mice with or without colitis. (**B**) Histograms depict the percentages of two distinct monocyte subsets, M1-type (Ly6C^high^CX3CR1^low^) and M2-type (Ly6C^low^CX3CR1^high^). Date are expressed as percentage of total monocytes (n = 6 in each group). *P < 0.05, ***P < 0.001 vs WT normal control group. ^##^P < 0.01, ^###^P < 0.001 vs WT with DSS induction group.

Because macrophages play a critical role in the pathogenesis of IBD, we then focused on macrophage polarization and function to investigate the mechanisms underlying the disease-ameliorating effects of SNX10 deficiency. Two populations of monocytes have been described in the murine circulation^19^. So-called “inflammatory” monocytes, characteristics by expression of Ly6C^high^CX3CR1^low^, represent a transient inflammatory stage from blood monocyte to tissue macrophage (M1 type). Another population of Ly6C^low^CX3CR1^high^ cells, the precursors of M2 macrophages, enters tissues constitutively via the activity of CX3CR1^19^. To determine whether the reduced inflammation in SNX10^−/−^ mice was due to the M2 polarization of macrophages, we first examined the monocytes subsets in peripheral blood of mice. The population of Ly6C^high^CX3CR1^low^ monocytes was similar between WT and SNX10 KO mice at steady state. However, it was significantly increased in WT, but not SNX10 KO mice after DSS treatment. Impressively, in both static state and after DSS treatment, Ly6C^low^CX3CR1^high^ monocytes were much higher in SNX10 KO mice than WT mice (Fig. 2A,B).

**Figure 3:**
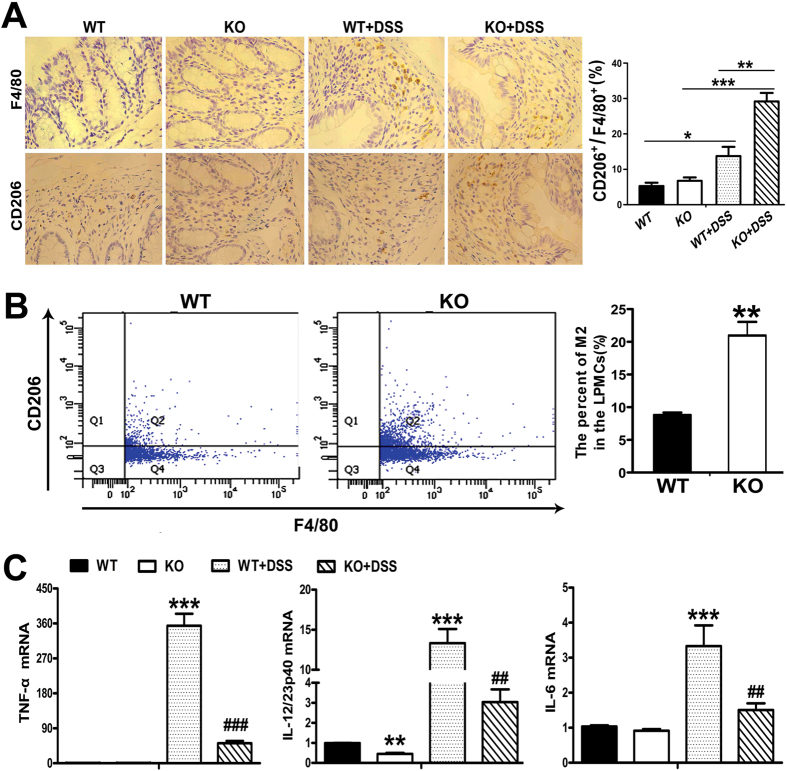
SNX10 deficiency increases the population of M2-type macrophages and decreases pro-inflammatory cytokine expression in colonic tissues. (**A**) Colonic tissue sections from normal control groups and DSS-induced colitis groups were stained respectively with anti-F4/80 and anti-CD206 antibodies to detect macrophage subpopulations (×10). The ratio between CD206^+^ and F4/80^+^ cells was calculated. (**B**) FACS analysis of macrophage subsets in colonic tissues of mice with DSS-induced colitis. The percentage of CD206+ cells in F4/80+ cells were calculated and shown as means ± SEM (n = 6 in each group). (**C**) The expression of pro-inflammatory cytokines in colonic tissues was determined by RT real-time -PCR. *P < 0.05, **P < 0.01, ***P < 0.001 vs WT normal control group. ^##^P < 0.01, ^###^P < 0.001 vs WT with DSS induction group.

Given the difference in precursor cells, we further characterized the macrophages infiltrating into colonic tissue during colitis. As shown in Fig. 3A, the number and staining intensity of F4/80-positive cells in colonic tissues of SNX10 KO mice with DSS-induced colitis were significantly lower than those in WT mice with DSS-induced colitis. There were more CD206 (M2 gene)-positive cells with stronger staining in the colonic tissue of SNX10 KO mice than WT mice (Fig. 3A). To further characterize the phenotype of macrophages infiltrating into colonic tissue, we isolated colonic lamina propria macrophages from the colon of WT and SNX10 KO mice with DSS treatment for fluorescence-activated cell sorting (FACS) analysis after CD206 and F4/80 antibody labeling. As shown in Fig. 3B, the percentages of F4/80^+^ CD206^+^ cells were significantly higher in SNX10 KO mice than in WT mice. Accordingly, the mRNA levels of TNF-α, IL-6, and IL-12/23 in colonic tissues of DSS-treated WT mice were increased, but were significantly reduced in SNX10 KO mice. Interestingly, in the normal condition, SNX10 deficient mice have lower levels of IL-12/23 in colonic tissues (Fig. 3C). These results suggest that SNX10 deficiency gives rise to an M2-type phenotype of monocytes and macrophages, which could contribute to the attenuation of the colonic inflammation induced by DSS.

### SNX10 deficiency polarizes macrophages towards an anti-inflammatory M2 phenotype

**Figure 4:**
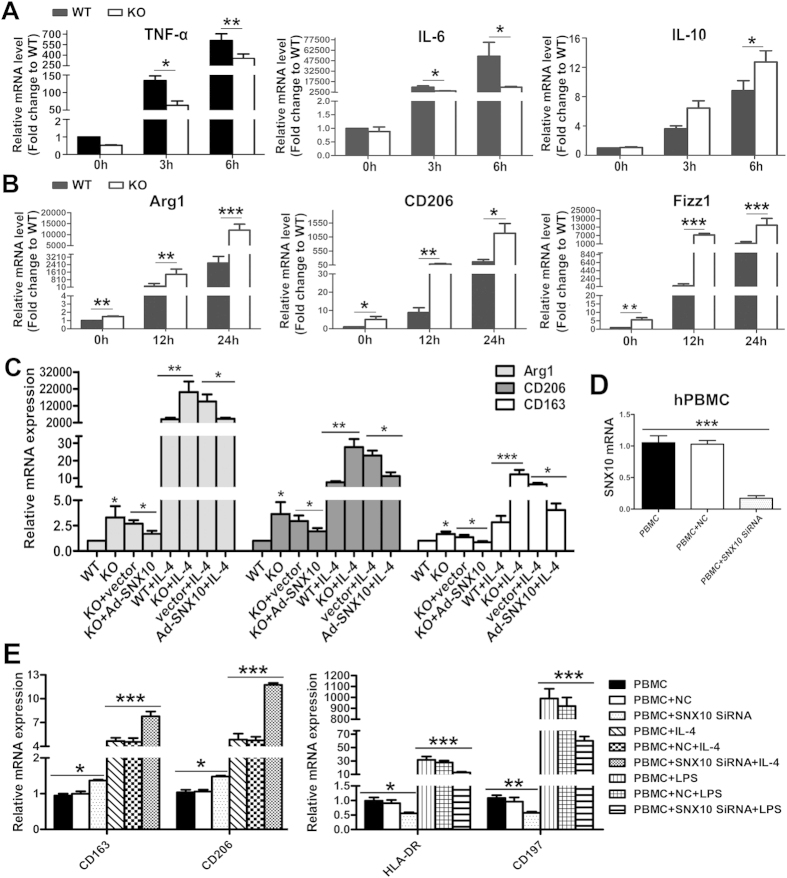
**SNX10 regulates the polarization of macrophages** ***in vitro***. (**A**) The levels of TNF-α, IL-6 and IL-10 mRNA in BMDMs from WT and SNX10 KO mice without or with the stimulation of 1 μg/ml LPS for 3 or 6 h. (**B**) The levels of Arg 1, CD263, Fizz1 mRNA in WT and SNX10 KO BMDMs without or with the stimulation of IL-4 for 12 or 24 h. (**C**) SNX10 KO BMDMs were transduced with adenoviral (Ad) expressing control or SNX10 plasmids for 24 h before stimulation with IL-4 for 24 h, followed by the examination of indicated M2 genes expression. (**D**) Human macrophages derived from PBMCs were treated without or with non-targeting control (NC) or SNX10 siRNA. The mRNA levels of SNX10 was measured by RT-quantitative PCR. (**D**,**E**) Human macrophages derived from PBMCs were treated with non-targeting control (NC) or SNX10 siRNA, followed by the stimulation of 20 ng/ml human IL-4 for 24 h (left), or 1 ug/ ml LPS for 6 h (right). The efficiency of siRNA was confirmed (**D**); and the mRNA levels of HLA-DR, CD197 (right), CD163 and CD206 (left) were measured (**E**). Data are means ± SEM. All experiments are repeated for three times at least.*P < 0.05, **P < 0.01, ***P < 0.001.

To confirm the influence of SNX10 deficiency on macrophage polarization, we used LPS to promote an M1 phenotype and IL-4 stimulation to induce an M2 phenotype. We found that SNX10^−/−^ bone-marrow-derived macrophages (BMDMs) failed to fully regulate M1 programming, which was indicated by impaired expression of TNF-α, IL-6 and IL-10 (Fig. 4A). Data also revealed that SNX10^−/−^ BMDMs responded strongly to IL-4, with a significant increase of hallmarks of M2 macrophages: Arg1, CD206 and Fizz1 (Fig. 4B). To further confirm these results, we took an adenoviral transduction approach to rescue SNX10 in SNX10^−/−^ BMDMs. As expected, Ad-SNX10 transduction in SNX10^−/−^ BMDMs decreased the mRNA levels of Arg1, CD206 and Fizz1 stimulated by IL-4, indicating inhibition of M2 polarization by SNX10 (Fig. 4C).

Consistent with the results from mice, SNX10 interference increased M2-type genes (CD163, CD206) and decreased M1-type genes (HLA-DR, CD197) in PBMCs-derived human macrophages (Fig. 4E). We also confirmed that SNX10^−/−^ human macrophages were more sensitive to IL-4 stimulation with increased hallmarks of M2 macrophages CD163 and CD206, but were relatively insensitive to LPS stimulation with diminished expression of M1-type genes HLA-DR and CD197 (Fig. 4E). The efficiency of SNX10 siRNA was confirmed by RT-quantitative PCR as shown in Fig. 4D.

Since SNX10 deficiency resulted in an M2-type polarization of macrophages, we determined whether the M2-type SNX10-deficient macrophages retained normal bacterial killing and phagocytic ability. As shown in Supplementary Fig. S1, there was no difference between SNX10^−/−^ and WT BMDMs in phagocytic capacity (Supplementary Fig. S1A). However, to our surprise, SNX10-deficient macrophages exhibit enhanced bactericidal activity (Supplementary Fig. S1B).

### The M2-polarized SNX10^−/−^ macrophages protect against DSS-induced colitis

**Figure 5:**
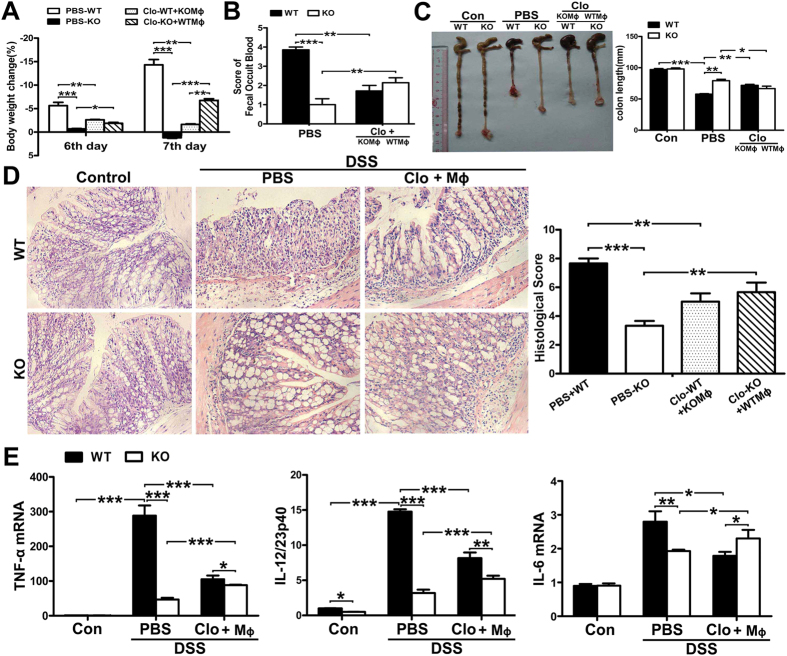
SNX10^−/−^BMDMs protect WT mice from DSS-induced colitis. Macrophages in WT and SNX10 KO mice were depleted by clodronate-liposomes (Clo) and then reconstituted with SNX10^−/−^ or WT BMDMs (KOM_ϕ_, WTM_ϕ_), respectively. PBS-liposomes (PBS) were used as the control. (**A**) Body weight changes of WT and SNX10^−/−^ mice in each group on day 6 and day 7 after DSS-induction. (**B**) Fecal occult blood of WT and SNX10 KO mice in each group at day 7 after DSS-induction was scored. (**C**) Representative images of colon. The length of colon in the indicated groups was statistically compared. (**D**) Representative H&E- stained sections of colon and histological assessment (original magnification, ×20). (**E**) Expression of pro-inflammatory cytokine mRNA in colonic tissues. Data are means ±SEM (n = 6 in each group). *P < 0.05, **P < 0.01 and ***P < 0.001.

To confirm that the protection of SNX10^−/−^ against DSS-induced colitis in mice was due to the M2 polarization of macrophages, we depleted macrophages from WT and SNX10^−/−^ mice using liposome-encapsulated clodronate, followed by transfer of SNX10^−/−^ or WT BMDMs. Macrophages labeled *in vitro* with red fluorescent dye IR783B-NHS were injected through the tail vein after *in vivo* depletion of macrophages to confirm that adoptive transfer was feasible. As shown in Supplementary Fig. S2, the adoptive macrophages were recruited into inflammatory colon. Administration of SNX10^−/−^ BMDMs significantly alleviated the loss of body weight at day 6 and 7 (Fig. 5A), reduced the score of fecal occult blood, and improved the shortness of colon in DSS-treated WT mice (Fig. 5B,C). Histological analysis showed that SNX10^−/−^ BMDMs significantly reduced colonic damage in WT mice with DSS-induced colitis (Fig. 5D). By contrast, transfer of WT macrophages into SNX10^−/−^ mice exacerbated the disease (Fig. 5A–D). These results were confirmed by the changes in mRNA levels of pro-inflammatory cytokines in the colon of macrophage-transplanted mice (Fig. 5E), suggesting a crucial role of M2-polarization of SNX10 deficient macrophages in protection against DSS-induced colitis.

## Discussion

IBD is an important cause of morbidity in most developed countries. The chronic, relapsing, and remitting or progressive inflammation of the gastrointestinal tract increase long-term risk of developing colon cancer^20^,^21^. Although the exact etiology and pathology of IBD is still poorly understood, available evidence suggests that dysregulation of the mucosal immune system due to excessive immune responses to normal microflora in the intestine plays a critical role in the pathogenesis of IBD, involving a wide range of molecules including cytokines^22^,^23^,^24^. Common treatments for inflammatory bowel diseases, such as corticosteroids and immunosupressive drugs, have undesirable side effects that could counteract their therapeutic benefits in the long term. In recent years, novel biological therapies designed against a specific inflammatory mediator, have emerged and found some of their first real clinical applications in treating IBD^25^. Infliximab, anti-tumor necrosis factor antibody, has proven efficacious in patients with refractory IBD. However, the immunogenicity and the risk for infectious complications hampered the long-term use of this drug. Therefore, new therapies created on alternative pathways in the inflammatory cascade would be desirable.

Macrophages play a crucial role in the pathogenesis of IBD and are regarded as meaningful target for drug development. Activated macrophage can be M1- or M2-polarized and its phenotype/function, to a large degree, is determined by the environment it enters^5^,^6^. Modulating the M1/M2 polarization status of macrophages can alter the course of the local inflammatory reaction in the colon, and this offers therapeutic potential for the management of IBD^7^,^8^. In our present study, SNX10 KO increased the population of M2-type monocytes and macrophages, and ameliorated the inflammation and tissue damage in colon with lower levels of proinflammatory cytokines TNF-α, IL-1β, IL-6, IL-12 and 23 mRNA. Consistent with the *in vivo* finding, *in vitro* study also showed SNX10 deficiency reduced LPS-induced M1 polarization which was indicated by impaired expression of TNF-α, IL-6 and IL-10, while promotes M2 polarization with a significant increase of hallmarks of M2 genes: Arg1, CD206 and Fizz1. The results from adenoviral transduction of SNX10 in SNX10 deficient macrophages confirmed the novel function of SNX10 in regulating macrophage polarization. By *in vivo* macrophage depletion and adoptive transfer approach, we further proved that the M2-polarized SNX10^−/−^ macrophages could significantly ameliorated DSS-induced colitis, suggesting that manipulating macrophage polarization targeting SNX10 may be an attractive new strategy for IBD treatment.

Macrophages execute diverse functions through phagocytosis, such as clearance of invading pathogens, resolution of inflammation, and maintenance of tissue homeostasis. Following internalization, the phagocytic particles undergo a maturation process through a series of increasingly acidified compartments, the phagosomes^26^. Both M1 and M2-polarized macrophages rely on the process of phagocytosis to exert their specific functions. A characteristic of M1 macrophages is the increased capacity to clear microbes and tumor cells, as well as the enhanced antigen-presenting efficiency through phagocytosis^27^. By contrast, M2 macrophages apply phagocytosis to maintain homeostatic functions, such as clearance of apoptotic cells and debris, tissue repair and remodeling, and suppression of inflammation^28^. Surprisingly, SNX10 deficient macrophages not only exhibit an M2 phenotype, but also gain increased microbicidal capacity. This specific character contributes to the protective function of SNX10 deficiency against colonic inflammation and pathological damage induced by DSS.

Patients with IBD are at a greater risk for osteopenia and osteoporosis. It was reported that more than 40% of patients with IBD have bone loss, which becomes the major complication affecting the quality of life and productivity^29^,^30^ Excessive osteoclast differentiation induced by bone marrow Th17 TNFα cells was recently identified to contribute to bone loss in IBD^31^. Osteoclasts are giant multinucleated, specialized bone resorbing cells that developed by the fusion of mononuclear precursors derived from the haematopoietic lineage. Pro-inflammatory cytokines particularly, RANKL and TNFα are considered as the most important incentives for osteoclast differentiation^30^. Following attachment to bone, osteoclasts are activated and form distinct and unique membrane domains that are crucial for the specialised bone resorbing function^32^,^33^. Previously, we found that SNX10 knockout impaired osteoclast maturation and bone resorption function through accelerating the degradation of nuclear factor NFATc1^14^. Here, we report that SNX10 regulates macrophage polarization and plays an essential role in inflammatory responses of IBD. It is of great interests for us to clarify whether SNX10 can provide a connection between inflammation and bone destruction in IBD is a great interest clarify in our future study.

In conclusion, our results demonstrate that SNX10 is a new regulator for macrophage M2 polarization, which is responsible for the protective effects against DSS-induced colitis in SNX10^−/−^ mice. Our study also provides evidence for Loss of SNX10 function can be a potential promising therapeutic strategy for IBD.

## Additional Information

**How to cite this article**: You, Y. *et al.* Sorting nexin 10 acting as a novel regulator of macrophage polarization mediates inflammatory response in experimental mouse colitis. *Sci. Rep.* **6**, 20630; doi: 10.1038/srep20630 (2016).

## Supplementary Material

Supplementary Information
