# Supplementary material for: Sorting nexin 10 acting as a novel regulator of macrophage polarization mediates inflammatory response in experimental mouse colitis
Source: Sci Rep. 2016 Feb 9;6:20630. doi: 10.1038/srep20630 (PMC4746623; doi:10.1038/srep20630)
Supplement: Supplementary Information [file srep20630-s1.pdf]

Sorting nexin 10 acting as a novel regulator of macrophage polarization mediates inflammatory response in experimental mouse colitis

Authors: Yan You[1], Chun Zhou[2], Dong Li[2], Zhong-Lian Cao[1], Weixing Shen[3], Wan-Zhen Li[2], Sulin Zhang[1], Bin Hu[1], Xiaoyan Shen[1,2]*

Author affiliation:

[1] Department of Pharmacology, School of Pharmacy, Fudan University, Shanghai, China

[2] Laboratory of Pharmacology and Toxicology, School of Pharmaceutical Sciences, Sun Yat-sen University, Guangzhou, China

[3] The Translational Medicine Research Center, Nanjing University of Traditional Chinese Medicine, Nanjing, China

* **Corresponding author:**

XiaoyanShen, M.D., Ph.D., Department of Pharmacology, School of Pharmaceutical Sciences, Fudan University, No. 826, Zhangheng Road, Pudong New Area, Shanghai 201203, China. Tel./fax: +86-21-51980182. E-mail address: shxiaoy@fudan.edu.cn.

Supplementary Figures and Figure legends

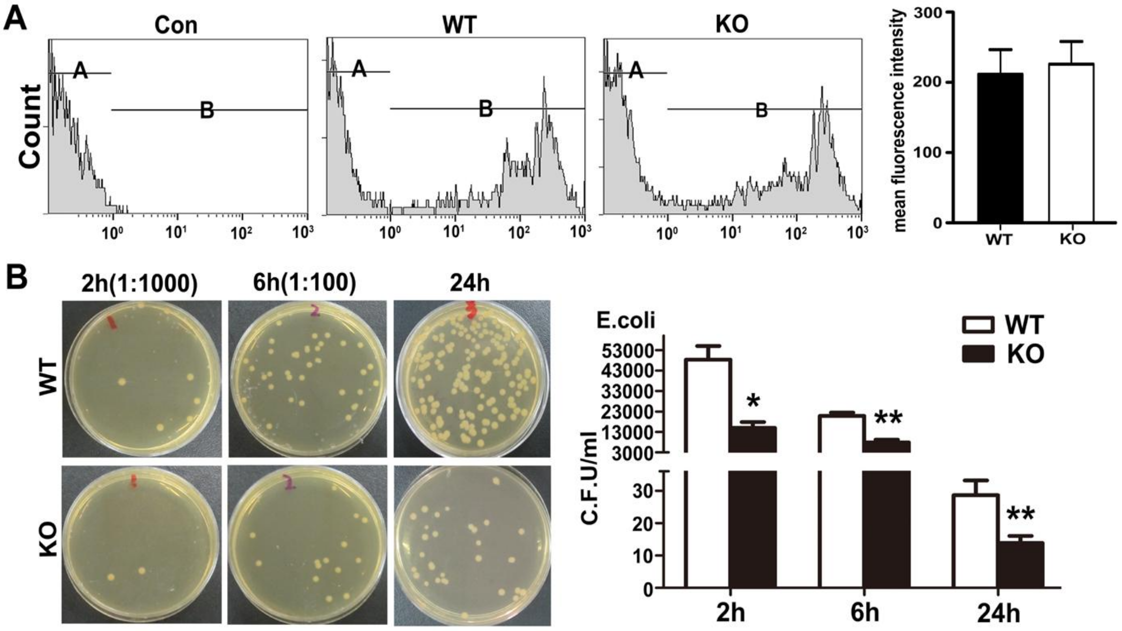

Figure S1 SNX10 deficiency enhances bacteria killing but not phagocytosis of macrophages. (A) BMDMs from SNX10 KO or WT mice were subjected to an ex vivo phagocytosis assay against bio-particles, followed by flow cytometry analysis and quantification. (B) BMDMs from SNX10 KO or WT mice were exposed to E. coli, intracellular viable bacteria were quantitated by colonies after plating the lysates of the macrophages on Luria-Bertani agar. *P < 0.05, ** P < 0.01 vs WT BMDMs.

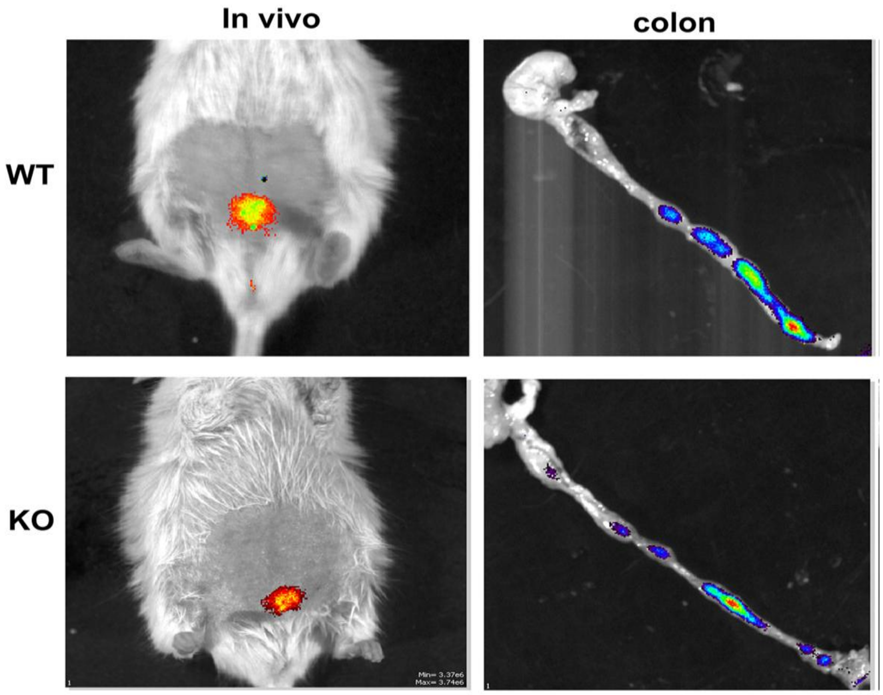

Figure S2 Tracking of transplanted macrophages in vivo during progression of colitis. The fluorescent-labeled WT and SNX10$^{-/-}$ macrophages were injected via tail vein at day 3 after received clodronate- or control PBS-loaded liposomes at day 1 and 2, followed by DSS induction. Whole body bioluminescence was visualized at day 7. Representative fluorescence photographs show the transplanted macrophages migrated to the inflammatory colon.
